# USP9X-mediated NRP1 deubiquitination promotes liver fibrosis by activating hepatic stellate cells

**DOI:** 10.1038/s41419-022-05527-9

**Published:** 2023-01-19

**Authors:** Jinqiu Zhao, Jie Bai, Fengling Peng, Chan Qiu, Yongguo Li, Li Zhong

**Affiliations:** 1 Department of Infectious Diseases, The First Affiliated Hospital of Chongqing Medical University, Chongqing, China; 2 Department of Gastroenterology, The Second Affiliated Hospital of Chongqing Medical University, Chongqing, China

**Keywords:** Liver diseases, Cell division

## Abstract

Liver fibrosis is a complex fibrotic process that develops early in the course of cirrhosis and is caused by chronic liver damage. The activation of hepatic stellate cells is primarily responsible for the fibrosis process. Studies show that NRP1 influences HSC motility and migration. However, whether NRP1 regulates HSC activation remains unknown. C57BL/6 male mice (6–8 weeks old) were intraperitoneally injected with 10% CCl_4_ in olive oil (5 μl/g body weight) every three days for four weeks to create an animal model of liver fibrosis. Control mice received olive oil (5 μl/g body weight). Different assays such as immunohistochemistry, immunostaining, Western blotting, qRT-PCR, immunoprecipitation, immunoprecipitation, and GST pull-down assays, and in vivo and in vitro ubiquitination assays were conducted. We found that NRP1 expression was significantly elevated both in mouse and human fibrotic livers, mainly in activated HSCs at the fibrotic foci. NRP1 promoted HSC activation via the cytokine TGF-β1, VEGFA, and PDGF-BB. Moreover, USP9X was found to be a critical deubiquitinating enzyme for the stability and high activity of NRP1 and NRP1 deubiquitination mediated by USP9X enhanced HSC activation and liver fibrosis. NRP1 deubiquitination mediated by USP9X enhances HSC activation, implying that targeting NRP1 or USP9X potentiates novel options in the treatment of liver fibrosis.

## Introduction

Liver fibrosis (LF) is a repair response to chronic hepatic injury which is described by an abnormal wound-healing process of which functional liver parenchyma is gradually replaced by fibrotic tissue with an increased accumulation of collagen-rich extracellular matrix (ECM) [1]. Most chronic liver diseases including alcoholic liver disease (ALD), viral hepatitis, and non-alcoholic fatty liver disease (NAFLD) are associated with LF [2]. LF is considerably correlated to severe liver complications such as hepatocellular carcinoma (HCC) and cirrhosis, which account for 3.5% of all annual fatalities globally [2].

About 10% of liver cells are nonparenchymal hepatic stellate cells (HSCs) which are the major source of ECM and play a major role in the progression of liver disease [3]. In the case of liver damage, HSCs are activated and produce ECM and many cytokines, as well as expressing α-smooth muscle actin (α-SMA) [4]. HSC phenotype transdifferentiation can be directed by transforming growth factor-β1 (TGF-β1), epidermal growth factor (EGF), platelet-derived growth factor (PDGF), and other cytokines [2, 5–7]. Emerging evidence suggest that inhibit HSC activation can effectively terminate or even cure LF [2, 8] which could serve as a candidate of antifibrotic approach.

Neuropilin-1 (NRP1) is a transmembrane receptor which contributes to the semaphorin and VEGF signaling cascades. NRP1 also contributes to angiogenesis, tumor development, vascular permeability, and immune functions [9]. In recent decades, NRP1 has received considerable attention as a component that enhances the penetration and pathogenicity of SARS-CoV-2 [10, 11]. The overexpression of NRP-1 in the liver has been reported to enhance liver injury in SARS-CoV-2 infection [12, 13]. In addition, studies have shown that NRP1 controls axonal guidance and cell migration [14, 15]. Cao et al. also found that NRP1 influences HSC motility and migration [16]. However, whether NRP1 regulates HSC activation and mechanisms underlying NRP1 dysregulation in HSCs remains elusive.

Proteolytic enzymes that cleave ubiquitin or ubiquitin-like proteins from target proteins or pro-proteins are known as deubiquitinating enzymes (DUBs) [17]. So far, more than one hundred DUBs have been identified in humans [17]. DUBs are related to cancer, neurological disorders, and long-term liver disease [18, 19]. Ubiquitin-specific peptidase 9X (USP9X), a member of the ubiquitin-specific protease (USP) family, is a highly conserved DUB [17]. Cell development, migration, polarity, and death are all affected by this DUB, which is well conserved [17, 20–22]. Remarkably, USP9X regulates the TGF-β signaling cascade by deubiquitination [22, 23]. However, there is a lack of clarity regarding the role of the USP9X protein in HSC activation and fibrogenesis.

In the present study, we determined the function of NRP1 in HSC activation as well as the mechanism that underpins its dysregulation in HSCs. We revealed that the NRP1 expression was significantly elevated in the liver of LF patients as well as in the mouse LF model. NRP1 is predominantly expressed in activated HSCs exist at the fibrotic foci. Moreover, we found that NRP1 promotes HSC activation, and NRP1 inhibition suppresses mouse primary HSC activation and protects mice from CCl_4_-induced LF. At the molecular level, we found that USP9X was a key DUB in regulating NRP1 ubiquitylation and its stabilization in HSCs. Thus, our findings provide new insights into the antifibrotic effects of NRP1 and USP9X.

## Methods

### Animals

6–8-week-old C57BL/6 male mice were obtained from The Chongqing Medical University’s Animal Experiment Center and housed in an environment free of pathogens with unlimited access to food and water. All procedures were approved by The Ethics and Medical Animal Care Committees of Chongqing Medical University. To create a liver fibrosis model, olive oil with carbon tetrachloride (CCl_4_) solution (5 μl/g body weight) was delivered intraperitoneally to mice three times per week for four weeks. The mice in the control group were given olive oil (5 μl/g body weight). GenePharma (1 × 10^13^ vg/mL, Shanghai) produced adenovirus (100 μl) with shRNA sequences targeting USP9X or nontargeting control sequences and administered it through the portal vein to establish USP9X’s participation in liver fibrosis. The shRNA sequence was 5′- GCCCAAATGAAGAAGTGACAA -3′.

### Primary HSC isolation and transfection

The isolation of primary HSCs (from around 3–6 mice) was carried out by collagenase-IV (0.5 mg/mL, Gibco) perfusion, and then density gradient centrifugation was performed with OptiPrepTM, as discussed previously [24, 25]. Trypan blue was used to evaluate the viability of HSCs, followed by determining cell purity using α-SMA (1:200, activated HSC marker) and glial fibrillary acidic protein (GFAP) (1:200, quiescent HSC marker). Cells were cultured in DMEM enriched with FBS (3%) at 37 °C and CO_2_ (5%) in a humidified environment. Primary HSCs were treated with PDGF-BB (20 ng/mL), TGF-β1 (5 ng/mL), and VEGFA (5 ng/mL) to identify the role of NRP1 in regulating HSC (MedChemExpress, USA). By western blotting (WB), qRT-PCR, and immunofluorescence staining. The transfection of ShRNA sequences or adenovirus vectors (Genechem. Shanghai, China) containing the cDNA of murine NRP1/USP9X was carried out into primary HSCs according to the manufacturer’s introduction.

### qRT-PCR evaluations

Total RNA was isolated By the RNA Simple Total RNA Kit (DP430, TIANGEN) according to the manufacturer’s introduction. The PrimeScript RT Reagent Kit (RR047A, TAKARA) was used to synthesize cDNA from the total RNA (in equal amounts) using a reverse transcriptase enzyme. The 2^-ΔΔCt^ technique was utilized to calculate gene expression and GAPDH was used to normalize it. The oligonucleotide primers utilized in this study are shown in Table 1.

**Table 1:** Characterization of the sequences of cDNA primers and shRNAs.

Sequences for shRNAs and cDNA primer		
Mouse gene	Forward (5′–3′)	Reverse (5′–3′)
NRP1	CCGATTCAGGACCATACAGG	TAGACCACAGGGCTCACCAG
α-SMA	GATCTCTATGCTAACAACGTCCTG	CTTCGTCGTATTCCTGTTTGC
Collagen I	CTGCCGTGACAAGCGAGTTC	GCTCACTCATGCGACTGAAACC
GAPDH	AACACGGAAGGCCATGCCA	GCATCCTGCACCACCAACTT
Human Gene		
NRP1	CTGGAATGTTGGGTATGGTGTCTGG	GAATGAGGTGCGGGTGGAAGTG
α-SMA	TGTGAAGCAGCTCCAGCTAT	CTTACAGAGCCCAGAGCCAT
Collagen I	CACCAATCACCTGCGTACAG	GCAGTTCTTGGTCTCGTCAC
GAPDH	CACTCCTCCACCTTTGACGC	CTGTTGCTGTAGCCAAATTCGT
USP9X shRNA #1	CGGCTTAACTTTCTTAGGTTT	
USP9X shRNA #2	GCCCAAATGAAGAAGTGACAA	
NRP1 shRNA #1	CCTGCTTTCTTCTCTTGGTTT	

### Antibodies

Antibodies utilized in this study are as follows: anti-α-SMA (GTX629702) from GeneTex; anti-USP9X (ab19879), anti-NRP1 (ab81321), Anti-Myc tag (ab9106), anti-Flag-Tag (ab205606), and anti-GST (ab92) from Abcam; anti-collagen I (91144S), anti-rabbit IgG (3900), anti-mouse IgG (5415), from Cell Signaling Technology; anti-GAPDH (10494-1-AP) from Proteintech.

### Western blotting (WB) and immunoprecipitation (IP)

The SDS-PAGE gel was employed to isolate proteins, followed by transferring onto the PVDF membrane. After blocking the membrane with 5% bovine serum albumin (BSA), primary antibodies (1:1000)were added, followed by rinsing and incubating with the secondary antibodies conjugated with the horseradish peroxidase (1:20000, BA1038/BA1039, Boster Biological Technology). An enhanced chemiluminescence kit (WBKlS0100, Millipore) was then employed to detect signals.

For immunoprecipitation, NETN buffer was utilized to prepare cell lysates. For each immunoprecipitation experiment, 400 μg cell extracts were subjected to immunoprecipitation with NRP1 or USP9X antibody overnight at 4 °C. The immunocomplex was precipitated with protein A/G agarose beads (Thermo Fisher Scientific) and evaluated by WB.

### GST pull-down and protein purification

For the GST pull-down test, bacteria expressing GST, GST-NRP1, GST-USP9X-WT, or GST-USP9X-C1566S were coupled to glutathione-Sepharose 4B beads (GE Healthcare) and incubated with Flag-USP9X WT, Flag-USP9X C1566S, or His-NRP1 expressed in murine HSCs for 2 hours at 4 °C. Post reaction, GST-binding buffer was utilized to rinse the complexes (at least 4 times), followed by elution in SDS-PAGE loading buffer (via boiling), and then WB analysis was conducted with the indicated antibodies.

### In vitro ubiquitination assays

Cells were transfected with various shRNAs as indicated for 72 h and then were incubated with MG132 (10 μM) 8 h and lysed with NETN buffer. Proteins were purified with IP with 400 μg cell extracts with S-protein agarose (Novagen)., After five times washing, the beads were eluted with SDS loading buffer, and then protein ubiquitinations were evaluated by WB.

The transfection of the primary mouse or murine HSCs was carried out with both His-NRP1 and HA-ubiquitin, followed by exposure to MG132 (10 μM) for 8 h to prepare ubiquitinated NRP1 as the substrate for the in vitro ubiquitination experiment. Ubiquitylated NRP1 was enriched by using an anti-His affinity column and then incubated with recombinant GST-USP9X-C1566S or GST-USP9X-WT proteins at 37 °C for 2 h in deubiquitylation buffer, and then protein ubiquitinations were examined by WB.

### Human tissue samples

The fibrotic liver biopsy samples were from the clinical patients in First Affiliation Hospital of Chongqing Medical University between June 2018 and July 2021. Normal liver tissues were from the patient with trauma underwent liver resection. This study was approved by the ethics committee of First Affiliation Hospital of Chongqing Medical University and all subjects signed a written informed consent form before sample collection. Specimens were fixed in paraformaldehyde (4%) after surgical liver resection for histological examinations or frozen in liquid nitrogen for mRNA and protein analyses.

### Histology, immunohistochemistry (IHC), and immunofluorescence

Samples were fixed with 4% paraformaldehyde and cut into 4µm-thick sliced. After deparaffinization and rehydration, antigen retrieval was achieved by heating in a pressure cooker for 5 min in 10 mM of sodium citrate (pH6) or 1 mM EDTA (pH8). Peroxidase activity was blocked by incubation in 3% H_2_O_2_ for 10 minutes. Sections were rinsed three times in PBS-T (0.1% Tween-20) and incubated in 5% BSA for 10 minutes. After the removal of the blocking solution, slides were placed into a humidified chamber and incubated overnight with primary antibodies in blocking buffer (5% BSA in PBS) and incubated overnight at 4 °C. After washing, slides were covered with SignalStain Boost IHC Detection Reagent (Cell Signaling Technologies, Boston, MA) for 30 min at room temperature. After washing two times with PBS-T, the Substrate-Chromgen Solution (VECTOR NovaRED, Substrate Kit, Vector Laboratories, Burlingame, CA) was applied, and slides were incubated 5–10 min and counterstained with Hemtoxylin. Images were acquired using a Zeiss Axiolab 5 Digital Lab Microscope (Carl Zeiss AG, Jena, Germany).

For immunofluorescence, After deparaffinization, rehydration and antigen retrieval, tissue slices were blocked with 5 % BSA before being incubated with the appropriate primary antibodies overnight at 4 °C. After washing, the slides were with secondary antibody (A32740 or A32723,), followed by counterstaining of the nuclei with DAPI. Images were acquired using a Zeiss Axiolab 5 Digital Lab Microscope (Carl Zeiss AG, Jena, Germany).

### Statistical analysis

All quantitative data were presented as the mean ± SEM of at least three independent experiments. Statistical analysis was performed by using SPSS software (version 19.0). Differences between unpaired groups were determined by using One-way ANOVA with parametric/nonparametric distributions. Unless otherwise stated, *P* < 0.05 was considered statistically significant.

### Reporting summary

Further information on research design is available in the Nature Research Reporting Summary linked to this article.

## Results

### NRP1 was significantly upregulated in fibrotic livers and essential for development of LF

**Fig. 1:**
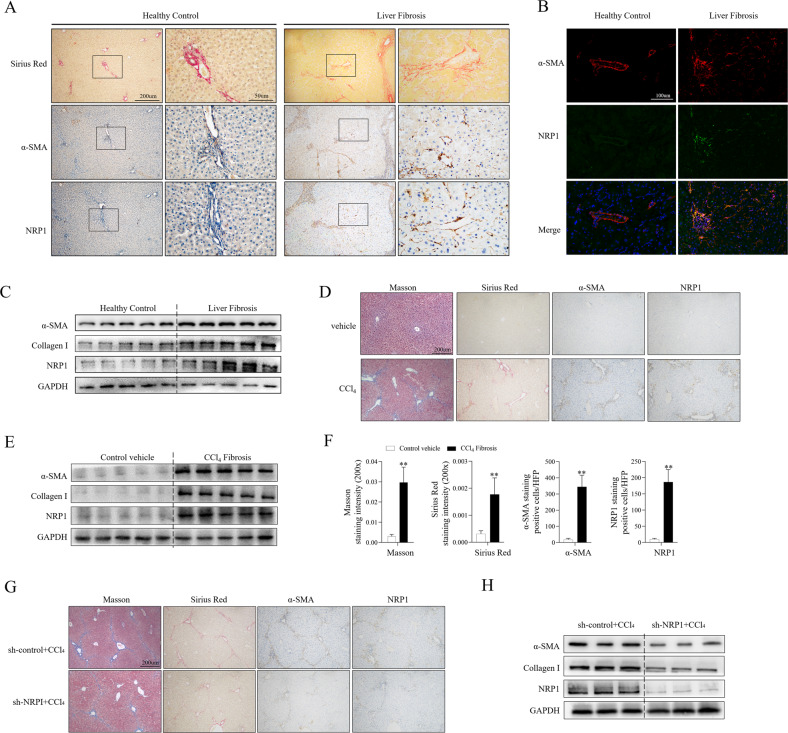
Elevated expression of NRP1 in fibrotic human and mouse liver tissues. **A** Immunohistochemical staining of Sirus Red, α-SMA and NRP1 in liver sections of healthy control and fibrotic human liver. **B** Immunofluorescence staining of α-SMA (red) and NRP1 (green) in liver sections as in (**A**). **C** WB analysis of α-SMA, collagen I and NRP1 in fibrotic and healthy human liver tissues. **D**–**F** Mice were treated with olive oil or carbon tetrachloride (CCl_4_) solution (5 μl/g body weight) via intraperitoneal injection three times a week for four weeks. Liver fibrosis was examined by Masson blue and Sirus Red staining (**D**). α-SMA, collagen I and NRP1 were evaluated by WB (**E**). Each group’s Masson positive and Sirius red staining region, and the number of NRP1^+^ and α-SMA^+^ cells in the given group was quantitatively evaluated (**F**). **G**, **H** CCl4 Mice were treated with adenovirus containing NRP1 interference sequence (sh-NRP1) or control sequence (sh-control) via vein injection. IHC (**G**) and WB (**H**) showed reduced liver fibrosis and decreased NRP1 and α-SMA expression in CCl4-treated mice after transfection with sh-NRP1. The graphs are represented as mean ± SD of at least three independent experiments. *P < 0.05; **P < 0.01.

To evaluate the role of NRP1in LF, the NRP1 expression was first examined in the liver tissues obtained from LF patients and healthy individuals (Fig. 1A–C). The NRP1 expression in the fibrotic tissues was found to be elevated compared with healthy controls (Fig. 1A–C). Furthermore, immunohistochemical staining (Sirius red staining) of fibrotic liver tissues indicated that NRP1 was largely present in the fibrous cords and septa, corresponding with the positive distribution of α-SMA, i.e., activated HSCs (Fig. 1A). Consistent with observations in human tissues, immunohistochemical staining on mouse fibrotic liver sections induced by CCl_4_ also displayed a substantial elevation in the expression of NRP1, particularly in the foci with broad fibrotic alterations (shown by positive Sirius red staining) (Fig. 1D). The results of qRT-PCR and WB also showed that NRP1 and fibrotic biomarkers, such as collagen I and α-SMA were elevated in CCl_4_-induced fibrotic livers of mice (Fig. 1E, F).

To determine whether NRP1 was required for the onset of LF, mice with CCl_4_-induced LF were infected with an adenovirus containing NRP1-interfering sequences (NRP1 shRNA). Interfering with NRP1 expression led to a marked remission of LF in CCl_4_-induced mice, as evidenced by a significant reduction in fibrotic areas (Masson and Sirius red staining) (Fig.1G) and decreased transcript expression levels of -SMA and collagen I (Fig. 1H).

### NRP1 was required for the mouse primary HSCs’ activation

**Fig. 2:**
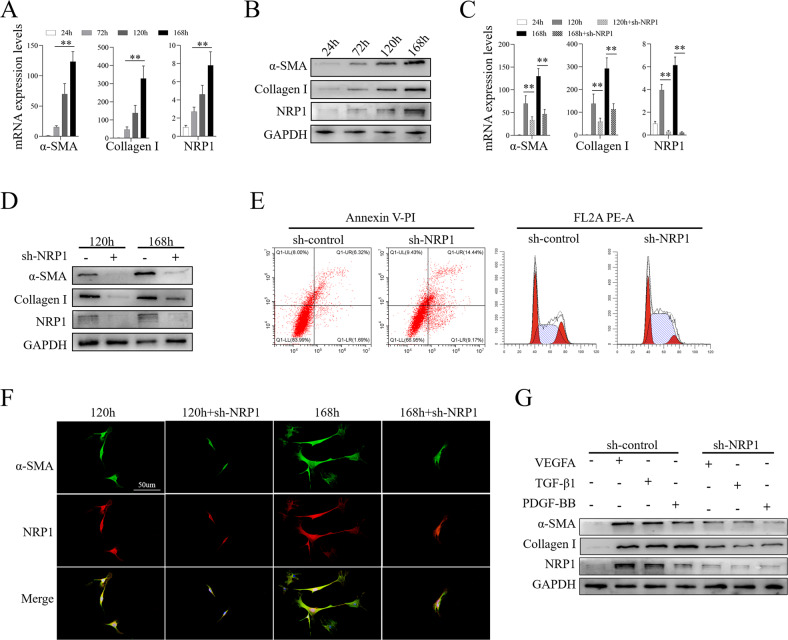
NRP1 was required for the mouse primary HSCs’ activation. **A**, **B**. HSCs isolated from mice were cultured in vitro for 24 h, 72 h, 120 h, or 168 h. **A** qRT-PCR analysis of α-SMA, collagen I and NRP1 in activated primary HSCs. **B** WB results of α-SMA, collagen I and NRP1 in activated primary HSCs. **C**–**G** Primary HSCs were transfected with NRP1 shRNA (sh-NRP1) or control sequence (sh-control) for 72 h, following 48 h of in vitro culture. The expression of NRP1, α-SMA, and collagen I was evaluated by qRT-qPCR (**C**) and WB (**D**). **E** Apoptosis and the cycle of primary HSC cells were detected by flow cytometry, after NRP1 shRNA or control shRNA transfection. The percentages of cell numbers in G0/G1, S and G2/M stages were 32.82%, 48.91% and 18.26% (sh-control), 27.40%, 66.20% and 6.40% (sh-NRP1), respectively. **F** The co-expression of α-SMA (green) and NRP1 (red) in HSCs was observed by immunofluorescence. **G** Primary HSCs were treated with PDGF-BB (20 ng/mL), TGF-β1 (5 ng/mL) and VEGFA (5 ng/mL) to accelerate activation, while transfected with NRP1 shRNA or control shRNA. The expression levels of collagen I and α-SMA were assessed by WB. The obtained data have been represented as mean ± SD. *P < 0.05; **P < 0.01.

The above date indicated the colocalization of NRP1 and α-SMA so we evaluated whether NRP1 promotes liver fibrogenesis by modulating the activity and function of HSCs by using primary HSCs. According to the results, NRP1 transcript and protein expression levels gradually elevated, which is associated with the elevation of HSC activating biomarkers, i.e., collagen I and α-SMA (Fig. 2A, B). NRP1 expression in primary hepatocytes was also examined, and the results showed that NRP1 was barely expressed in primary hepatocytes (Fig. S1). To further confirm whether NRP1 is required for HSCs activation, NRP1 expression interfered with adenovirus (sh-NRP1) in primary HSCs and the results indicated that NRP1 knockdown significantly decreased the collagen I and α-SMA expression levels, indicating that NRP1 knockdown inhibited HSC activation (Fig. 2C, D). We further analyzed cell apoptosis and cell cycle by using flow cytometry and the results showed that NRP1 interfering in primary HSCs increased apoptosis and the proportion of S-phase cells (Fig. 2E). We conducted immunofluorescence of primary HSCs and found that the NRP1 and α-SMA expression steadily increased over time, but NRP1 knockdown resulted in a significant reduction of α-SMA expression (Fig. 2F). To further examined whether NRP1 plays a role in the fibrogenic response generated by cytokines including TGF-β1, PDGF-BB, and VEGFA in HSCs (Fig. 2G). HSCs activation (as evidenced by collagen I and α-SMA levels) was markedly abrogated by NRP1 knockdown (Fig. 2G).

### Direct interaction between USP9X and NRP1

**Fig. 3:**
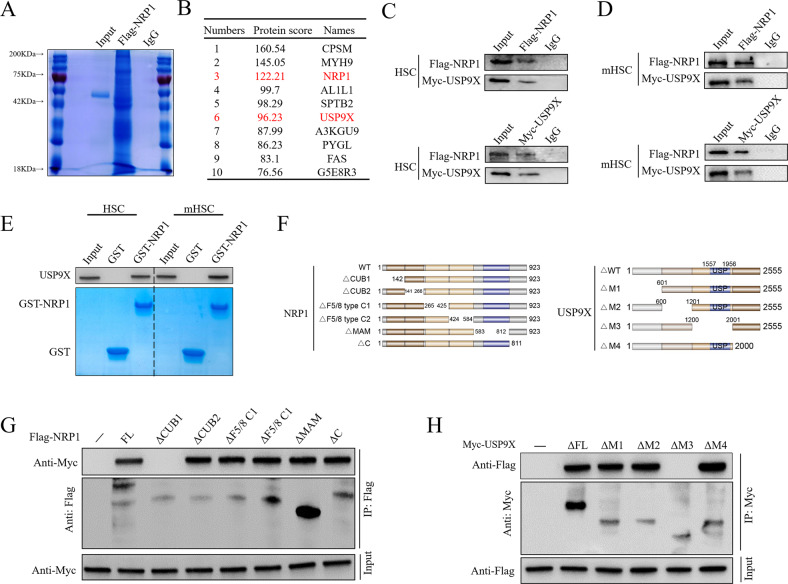
Direct interaction between USP9X and NRP1. **A** Mouse primary HSCs were extracted and transfected with Flag-NRP1 for 24 h, and then CO-IP (with Flag beads) was used to evaluate cell lysates, followed by IB with antibodies against Flag. Post-staining with coomassie blue, proteins were collected, followed by identification through LC-MS/MS. **B** In mouse primary HSCs, LC-MS/MS analysis revealed NRP1 interacting proteins. Each peptide’s name and the number of peptides are mentioned. **C** IP was conducted to evaluate cell lysates from primary mouse HSCs using antibodies against USP9X and NRP1, followed by IB evaluation. As an isotype control, IgG was utilized. **D** IP was conducted to evaluate cell lysates from murine HSCs using antibodies against USP9X and NRP1, followed by IB evaluation. As an isotype control, IgG was utilized. **E** GST or GST-NRP1 coupled glutathione-Sepharose beads were used to incubate cell lysates from primary mouse HSCs or murine HSCs. Proteins that remained on Sepharose were then evaluated by IB with the appropriate antibodies. SDS-PAGE and Coomassie blue staining were used to examine recombinant GST-NRP1 isolated from bacteria. **F** NRP1, USP9X, and their several deletion mutants are depicted schematically. **G** The cotransfection of HEK293T cells was carried out with Myc-USP9X and Flag-NRP1 or its deletion mutants for 24 h, and then cell lysates were evaluated through IP with Flag beads followed by IB with antibodies against Flag and Myc. **H** The cotransfection of HEK293T cells was carried out with Flag-NRP1 and Myc-USP9X or its deletion mutants for 24 h, and then the evaluation of cell lysates was carried out through IP with His beads followed by IB with antibodies against Flag and Myc.

To explore how NRP1 was upregulated in liver fibrosis, we transfected Flag-NRP1 in mouse primary HSCs, followed by Co-IP and LC-MS/MS proteomic analysis to examine NRP1-interacting proteins (Fig. 3A). The results revealed that deubiquitinase USP9X is one of the potential NRP1 binding proteins (Fig. 3B). To confirm this finding, we performed Co-IP to examine whether USP9X interacts directly with NRP1 (Fig. 3C, D). The results showed interactions between NRP1 and USP9X in both primary mouse HSCs and murine HSCs (Fig. 3C, D)., We further performed in vitro GST pull-down assay by adding purified GST-NRP1 into cell lysates from primary mouse HSCs or murine HSCs. we found that USP9X could bind to GST-NRP1 rather than to GST alone, which indicated direct interaction between USP9X and NRP1 (Fig. 3E). We further generated truncated mutants of Myc-USP9X and Flag-NRP1 to investigate interacting sites and define the minimal critical areas necessary for their binding (Fig. 3F). The results indicated that NRP1’s CUB1 domain (amino acids 1–141) and USP9X’s middle sequences (amino acids 1201–2000) are both required for direct interactions (Fig. 3G, H).

### USP9X was primarily responsible for NRP1 stability

**Fig. 4:**
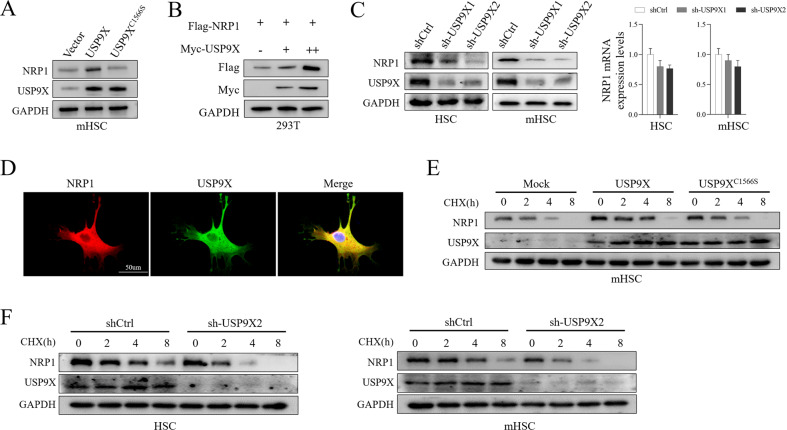
USP9X was primarily responsible for NRP1 stability. **A** Murine HSCs were transfected with a plasmid (control, USP9X WT or USP9X C1566S mutant), and cell lysates were evaluated through IB with antibody against NRP1. **B** The transfection of HEK293T cells was carried out with a high level of Myc-USP9X for 24 h, followed by evaluation of cell lysates through IB with antibody against Flag. **C** Primary mouse HSCs and murine HSCs were transfected with 2 independent USP9X shRNA for 72 h, and then USP9X and NRP1 were analyzed using WB and qRT-PCR. **D** Immunofluorescence detection of USP9X and NRP1 cells expressing location. **E** Transfection of murine HSCs was carried out with adenovirus (control, USP9X WT or USP9X C1566S mutant), followed by exposure to CHX (100 μg/ml), and collection at the given times, and then IB evaluations were carried out with antibodies against NRP1 and USP9X. **F** Primary mouse HSCs or murine HSCs (stably expressing control shRNA or USP9X shRNA) were exposed to CHX (100 μg/ml), followed by collection at the given time, and then IB analysis was carried out with antibodies against NRP1 and USP9X.

We further examined whether USP9X influences NRP1 stability, murine HSCs were transfected with WT USP9X or the catalytically inactive mutant C1566S USP9X and examined NRP1 protein level (Fig. 4A). NRP1 protein levels were elevated by WT USP9X but not the C1566S mutant (Fig. 4A). Similar results were observed when co-transfected Myc-USP9X with flag tagged NRP1 into HEK293T cells (Fig. 4B). These data suggested that USP9X stabilized NRP1 in a DUB activity-dependent manner.

To further assess whether USP9X is required for maintaining NRP1 stability, we knocked down USP9X in primary mouse HSCs and murine HSCs and examined NRP1 protein levels (Fig. 4C). USP9X knockdown resulted in a dramatic reduction of NRP1 protein but not the NRP1 transcriptional levels (Fig. 4C). Immunofluorescence staining showed the colocalization of NRP1 and USP9X in primary mouse HSCs (Fig. 4D). We measured NRP1 half-life in cells overexpressed USP9X (Fig. 4E). We observed prolonged NRP1 protein half-life when the WT USP9X protein was overexpressed, but not in the C1566S mutant (Fig. 4E). Similarly, halflife of NRP1 was decreased when USP9X protein was knocked down (Fig. 4F).

### USP9X induced deubiquitination of NRP1

**Fig. 5:**
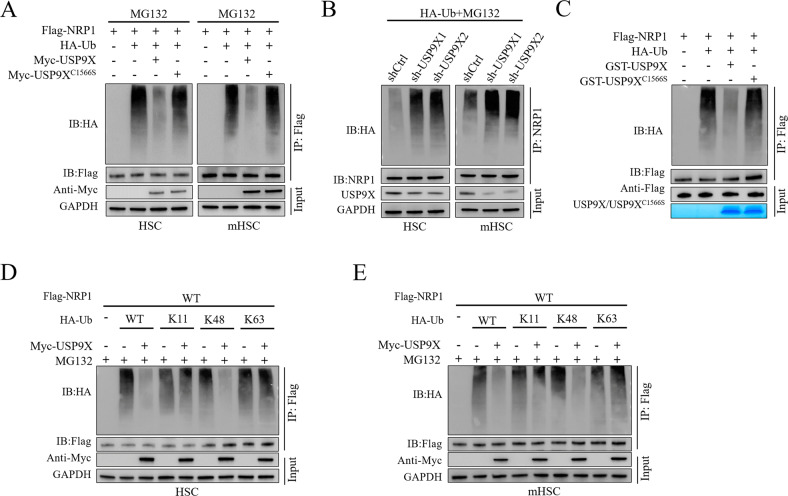
USP9X deubiquitinates NRP1. **A** Primary mouse HSCs and murine HSCs were co-transfected with Flag-NRP1HA-ubiquitin (HA-Ub), and Myc-USP9X WT or Myc-USP9X C1566S for 24 h, cells were then exposed to MG132 (10 μM) for 8 h. Ubiquitination level of NRP1 was evaluated IP with Flag beads and IB analysis antibodies against HA a (**B**). Primary mouse HSCs and murine HSCs were transfected with HA-Ub for 24 h, followed by USP9X knockdown with shRNA targeting USP9X for 72 h. Cells were then exposed to MG132 (10 μM) for 8 h. Ubiquitination level of NRP1 was evaluated IP with Flag beads and IB analysis antibodies against HA (**C**). GST-USP9X C1566S or GST-USP9X WT coupled to glutathione-Sepharose beads were treated with unubiquitylated or ubiquitylated Flag-NRP1, followed by Flag-NRP1 evaluation by IP with Flag beads and then IB evaluation was carried out with antibodies against HA and Flag. SDS-PAGE and Coomassie blue staining were used to examine recombinant GST-USP9X or GST-USP9X C1566S. **D**, **E** Murine HSCs (**D**) or primary mouse HSCs (**E**) were co-transfected with Flag-NRP1, Myc-USP9X, in the absence or presence of HA-Ub, HA-Ub K11-only, K48-only or K63-only plasmids, NRP1 ubiquitylation linkage was determined by IP with Flag and IB with HA.

We further assessed whether USP9X catalyzed deubiquitination of NRP1 by coexpressing NRP1 with USP9X WT or the C1566S mutant in primary mouse HSCs and murine HSCs cell lines and compared ubiquitination levels (Fig. 5A). NRP1 protein was shown to be highly ubiquitinated in the presence of MG132, co-expression of WT USP9X but not C1566S mutant nearly eliminated NRP1 ubiquitination (Fig. 5A). In contrast, downregulation of USP9X by two independent shRNA significantly elevated ubiquitylation of NRP1 in primary mouse HSCs and murine HSCs (Fig. 5B).

To further examine whether NRP1 was a direct deubiquitinated substrate of USP9X, we performed in vitro deubiquitination assay by incubating purified USP9X C1566S and USP9X WT and incubated with NRP1 under cell-free conditions and examined NRP1 ubiquitination (Fig. 5C). The results indicated thatGST-USP9X WT but not GST-USP9X C1566S disassembles NRP1 ubiquitin molecules in vitro (Fig. 5C). Polyubiquitin chains have been found to be linked in two distinct ways so far (K48- or K63- linked chains). The K48-linked ubiquitin chain acts as the primary target signal for the proteasomal degradation of proteins, whereas the K63-linked ubiquitin chain is associated with diverse cellular activities working independently of the proteasome-mediated degradation cascade. Therefore, we further investigated which polyubiquitin alterations on the NRP1 protein were influenced by USP9X. Our results indicated that in both mouse primary HSCs and HSCs, USP9X successfully disintegrated K48-linked polyubiquitylation of NRP1 but had no impact on K63-linked polyubiquitylation of NRP1 (Fig. 5D, E).

### Inactive USP9X attenuated mouse LF via destabilizing NRP1

**Fig. 6:**
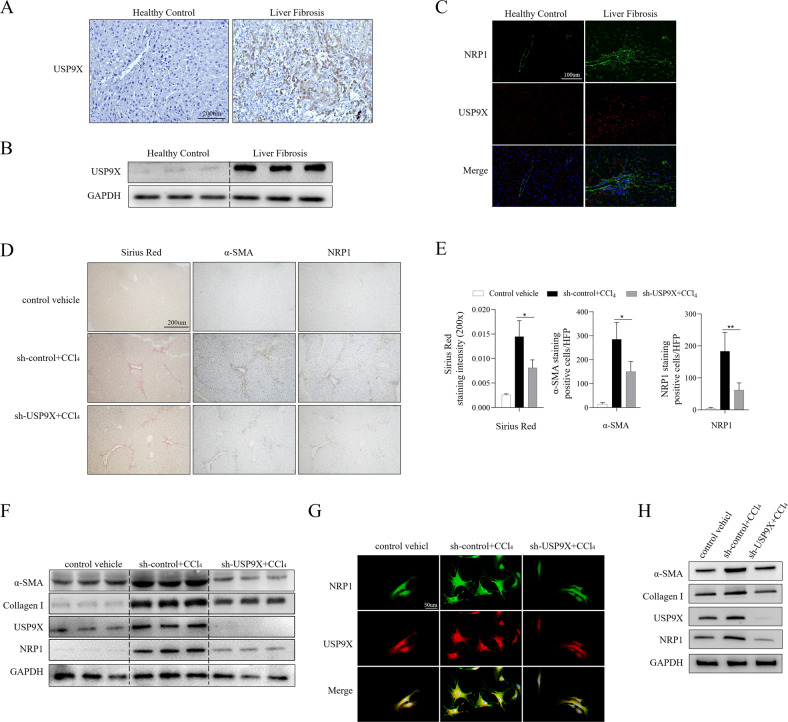
Inactive USP9X attenuates mouse LF via destabilizing NRP1. **A** Immunohistochemical staining of USP9X in liver sections of healthy control and fibrotic human liver. **B** WB analysis of USP9X in fibrotic and healthy human liver tissues. **C** Immunofluorescence staining of USP9X (red) and NRP1 (green) in liver sections as in (**A**). **D**–**G** Mice were treated with olive oil or carbon tetrachloride (CCl_4_) solution (5 μl/g body weight) via intraperitoneal injection three times a week for four weeks. CCl4-induced LF mice were then infected with adenovirus containing USP9X interference sequence (sh-USP9X) or control sequence (sh-control) via vein injection. **D** Liver fibrosis was examined by Sirus Red staining. **E** The Sirius red staining region quantitative analysis and the number of NRP1+ and SMA+ cells in the underlined group in each group are shown. **F** WB analysis of USP9X, NRP1, α-SMA, and Collagen I expression in the liver of the CCl4-induced plus USP9X knockout group and the CCl4-induced group alone. **G** HSCs isolated from mice were cultured in vitro for 120 h. Immunofluorescence staining of USP9X (red) and NRP1 (green) in HSC. **H** WB results of USP9X, NRP1, α-SMA, and Collagen I expression of primary HSCs isolated from CCl_4_-induced plus USP9X knockdown group, compared to those from CCl_4_ induction group alone. The obtained data have been indicated as mean ± SD. *P < 0.05; **, *P* < 0.01.

To confirm the role of the USP9X/NRP1 axis in LF, we first evaluated UPS9X expression in the hepatic tissues obtained from LF patients and healthy individuals by immunohistochemical staining, WB, and immunofluorescence staining (Fig. 6A–C). All results indicated a significant elevation in the expression of USP9X fibrotic than in non-fibrotic tissues. We further assess the role of the USP9X/NRP1 axis in LF in vivo (Fig. 6D–F). Adenovirus carrying the USP9X interference sequence (sh-USP9X) was injected into the splenic vein of mice injected with CCl_4_. We evaluated fibrosis by Masson and with Sirius red staining. CCl_4_-treated livers showed prominent disordered architecture with the formation of septa between nearby vascular structures and signs of fibrosis involving certain features of parenchymal nodules. The sh-USP9X-CCl4-treated group had a significantly lower degree of diaphragm development (Fig. 6D, E). In addition, lower expression levels of NRP1 as well as fibrotic proteins α-SMA and collagen I were observed following interference with USP9X (Fig. 6F). Immunofluorescence staining of primary HSCs revealed that HSCs in the sh-USP9X-CCl4-treated group exhibited low NRP1 expression and displayed more quiescent morphological features than the fibrocyte phenotype (Fig. 6G). WB analysis further confirmed that USP9X knockdown resulted in a decrease of protein expression of α-SMA, Collagen I, and NRP1 in primary mouse HSCs (Fig. 6H).

## Discussion

**Fig. 7:**
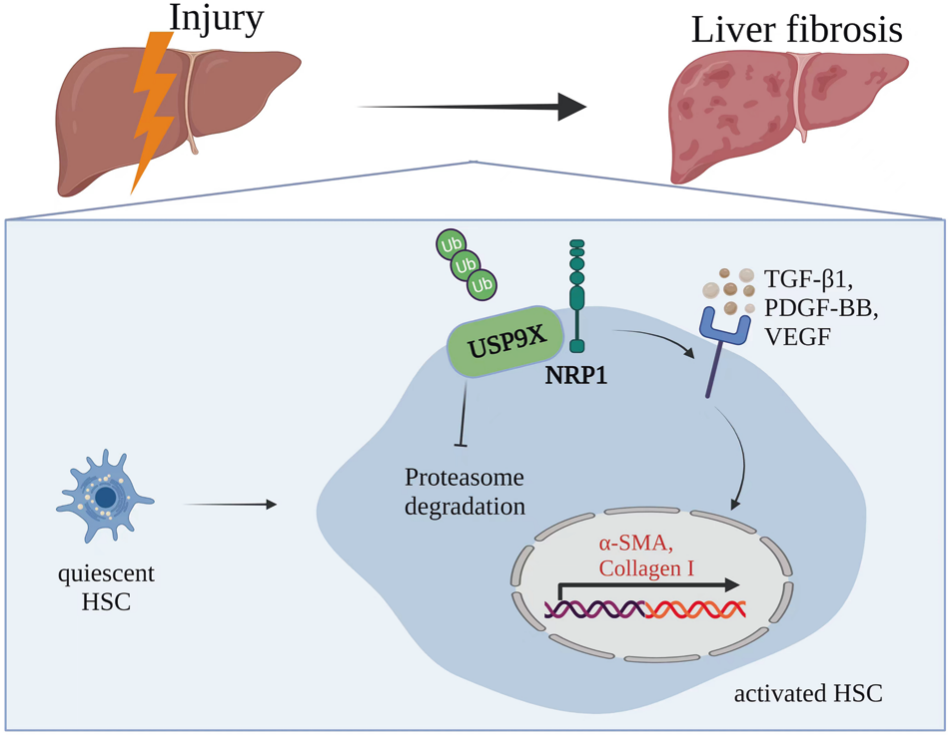
The molecular mechanism underlying USP9X-mediated NRP1 deubiquitination enhances liver fibrosis by triggering HSCs. NRP1 is stabilized and upregulated by the deubiquitinase USP9X. Increased NRP1 levels promote HSC activation and the secretion of elevated levels of ECM and inflammatory factors through cytokine TGF-β1, VEGF and PDGF-BB.

In the present study, We reported that NRP1 expression was elevated in both mouse and human fibrotic livers. Inactivation of NRP1 prevented Ccl4-induced liver fibrosis in vivo. We further revealed that NRP1 promoted HSC activation via the cytokine TGF-β1, VEGFA, and PDGF-BB. At the molecular level. We found that USP9X was a critical deubiquitinating enzyme which primarily responsible for the NRP1 stability and NRP1 deubiquitination mediated by USP9X enhanced HSC activation. Inactive USP9X ameliorated liver fibrosis in vivo via destabilizing NRP1. Our data thus shed new lights into mechanisms underlying stellate cell activation and liver fibrosis and targeting USP9X/NRP1 might serve as potential target for liver fibrosis (Fig. 7).

Hepatic stellate cells (HSCs) are one of the key cell types implicated in hepatic fibrosis [4]. HSCs are activated and differentiated into myofibroblasts during liver fibrogenesis. It is well established that HSCs activaition has a critical contribution to hepatic fibrogenesis [8]. The activation of HSCs is a multistep process that involves multiple functional and morphological modifications. Various events lead to the activation of HSCs, such as an elevated level of oxidative stress, inflammation, and paracrine signaling via TGF-1β, PDGF-BB, and VEGF. HSC activation inhibition is a potentially promising target for LF [26]. Our data clearly indicated the critical role of NRP1 in HSCs activation via cytokine TGF-β1, VEGFA, and PDGF-BB but the mechanisms by which NRP1 promotes HSCs activation remain unlear.

NRP-1 is best recognized for its activity as a cell surface receptor and its participation in cellular signaling [27]. Receptors for signaling molecules such as semaphorins, VEGF (specifically the VEGF-A isoform), TGF-β, plexins, and integrins are found on NRP-1’s surface. Several pieces of research indicated the vital role of NRP1 in liver diseases including liver cirrhosis and liver cancer [16, 28, 29]. Herein, we observed that NRP1 has an elevated expression in human and mouse LF tissues. Moreover, our data revealed that interfering with NRP1 expression suppressed the activation of mouse primary HSCs, further supporting our hypothesis that NRP1 is a profibrotic factor and could serves as therapeutic target of LF. Mechanistically, the obtained data reveal that NRP1 stimulates TGF-β1, VEGFA, and PDGF signaling. According to prior research [16], NRP-1 functions as a co-receptor for PDGF and TGF-, which is consistent with our findings. NRP-1 also promotes the myofibroblast phenotype by mediating divergent R-Smad signaling [30]. Hence, the underlined data suggested that NRP1 target multiple growth factors signaling pathways, and eventually contributes to HSCs activation and LF. Targeting of NRP1 inhibition overcomes HSCs activation and LF progression significantly.

The current study provides evidence supporting the extraordinary significance of NRP1 in stimulating HSC activation. However, there is a lack of clarity regarding their underlying upstream regulatory mechanisms. The current study’s Co-IP and LC-MS/MS proteomic analysis showed that USP9X could bind to NRP1. This study also confirmed that their mutual interactions require the CUB1 domain (1–141 amino acids) of NRP1 and the middle sequences (1201–2000 amino acids) of USP9X. USP9X is a crucial deubiquitinating enzyme that stabilizes various substrates and directly regulates numerous proteins’ functions. USP9X has been linked to endocytosis regulation, a remarkable function of NRP1. USP9X has been shown to regulate EGFR endocytosis in tumor cell lines by deubiquitinating its endocytic adaptor Eps15 and ubiquitin ligase Itch [31].

In contrast, USP9X also binds directly to receptors such as ErbB2/HER2, thereby regulating their intracellular localization and transport [32]. Given the preceding findings, we postulate that USP9X performs its function by deubiquitinating NRP1. In accordance with our hypothesis, we investigated the specific deubiquitination effects of USP9X on NRP1. NRP1 underwent more ubiquitination modifications and was thus more susceptible to protein degradation without USP9X. These findings indicate that deubiquitinase USP9X is a novel upstream regulator interacting with and stabilizing NRP1.

We further investigate the function of the USP9X/NRP1 signal in LF and discovered that the expression of USP9X was upregulated in LF tissues. Moreover, USP9X expression inhibition attenuated HSC activation and liver fibrosis, accompanied by low NRP1 expression. NRP1 has been confirmed as a novel substrate for the deubiquitinating enzyme USP9X. USP9X induced HSC activation and LF development in an NRP1-dependent manner, as shown by these results.

In conclusion, NRP1 contributes to HSC activation and liver fibrosis. We further showed that USP9X is a critical deubiquitinating enzyme for the stability and high activity of NRP1. Thus, targeting NRP1 or USP9X for treating liver fibrosis could bring up new therapeutic options.

## Supplementary information

Reporting Summary

Supplemental Figure 1 The NRP1 expression in activated primary hepatocytes were assessed by immunofluorescence (A) and WB (B).

Original Data File

Original Data File

Original Data File

Original Data File

Original Data File

Original Data File

SUPPLEMENTAL figure1

EDITORIAL CERTIFICAT

## Data Availability

Some or all data, models, or code generated or used during the study are available in a repository or online in accordance with funder data retention policies (Provide full citations that include URLs or DOIs.)
