# Supplementary material for: USP9X-mediated NRP1 deubiquitination promotes liver fibrosis by activating hepatic stellate cells
Source: Cell Death Dis. 2023 Jan 19;14(1):40. doi: 10.1038/s41419-022-05527-9 (PMC9849111; doi:10.1038/s41419-022-05527-9)
Supplement: Supplementary file 2 — Supplemental Figure 1 The NRP1 expression in activated primary hepatocytes were assessed by immunofluorescence (A) and WB (B). [file 41419_2022_5527_MOESM2_ESM.docx]

**Supplemental Figure 1** The NRP1 expression in activated primary hepatocytes were assessed by immunofluorescence (A) and WB (B).
