# Supplementary material for: USP9X-mediated NRP1 deubiquitination promotes liver fibrosis by activating hepatic stellate cells
Source: Cell Death Dis. 2023 Jan 19;14(1):40. doi: 10.1038/s41419-022-05527-9 (PMC9849111; doi:10.1038/s41419-022-05527-9)

Figure B

GAPDH

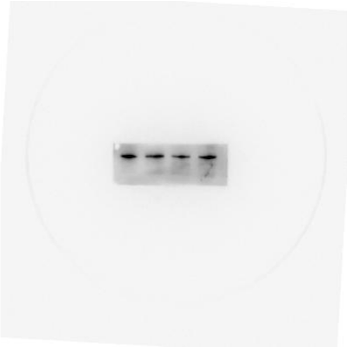

GAPDH2

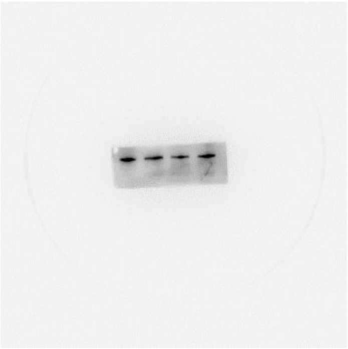

α-SMA

CollagenI

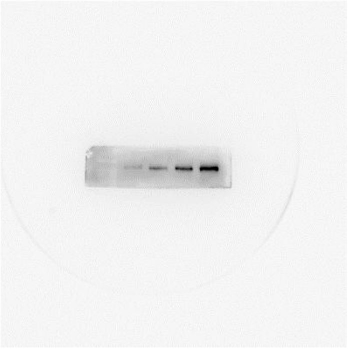

Nrp1

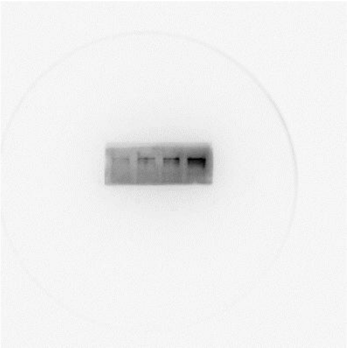

Figure D

GAPDH

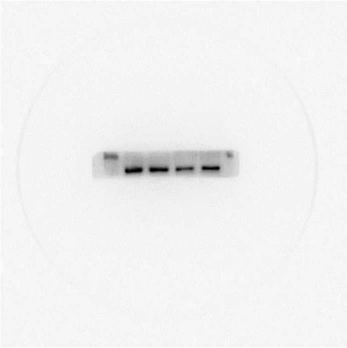

α-SMA

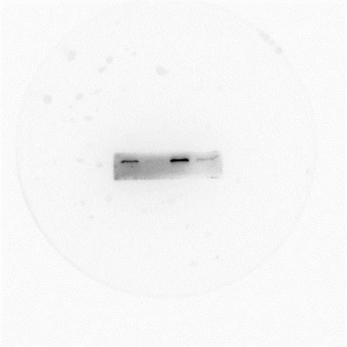

CollagenI

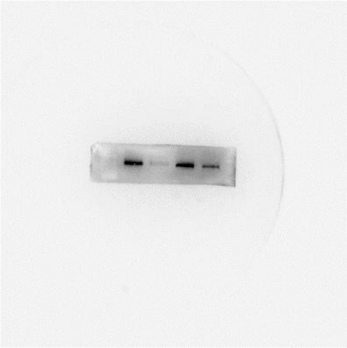

Nrp1

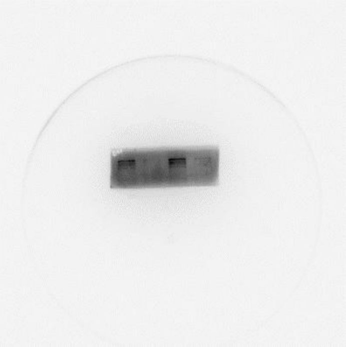

Figure G

GAPDH

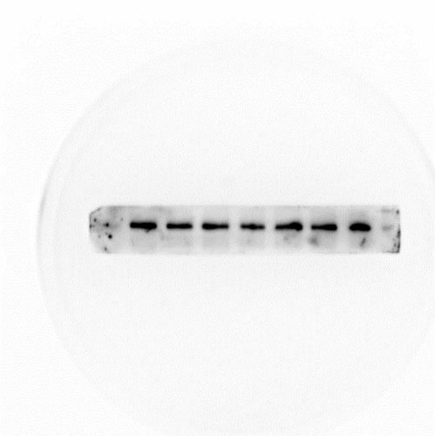

α-SMA

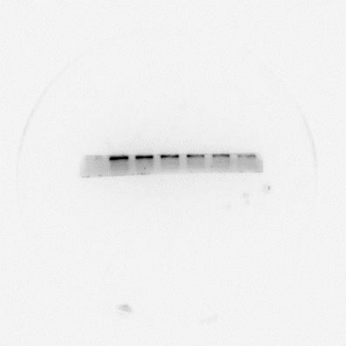

CollagenI

Nrp1

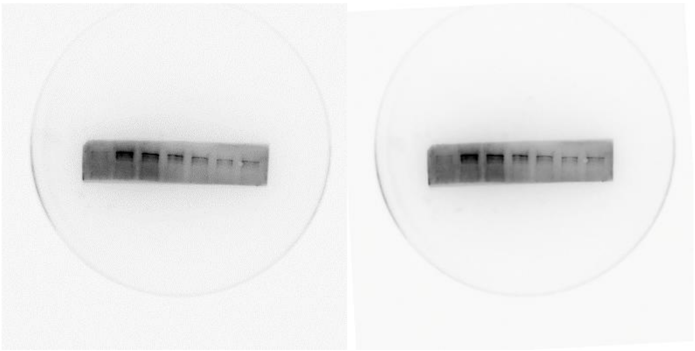

Supplement: Supplementary file 4 — Original Data File [file 41419_2022_5527_MOESM4_ESM.pdf]
