# Supplementary material for: USP9X-mediated NRP1 deubiquitination promotes liver fibrosis by activating hepatic stellate cells
Source: Cell Death Dis. 2023 Jan 19;14(1):40. doi: 10.1038/s41419-022-05527-9 (PMC9849111; doi:10.1038/s41419-022-05527-9)

Figure4 A

GAPDH(1,2)

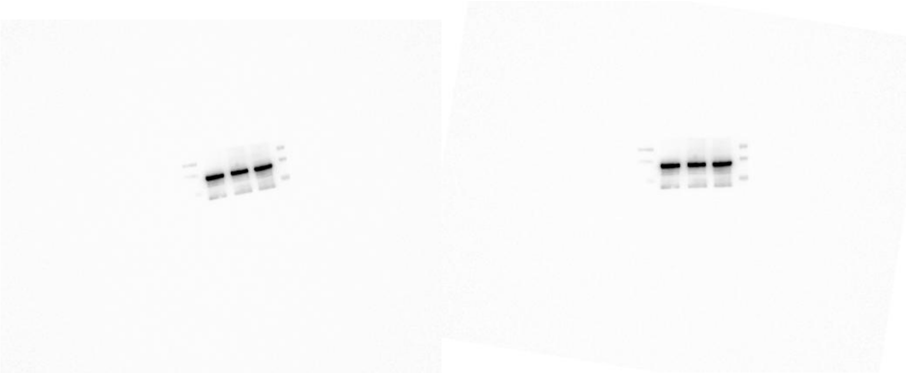

Nrp1

Uxp9x(1,2)

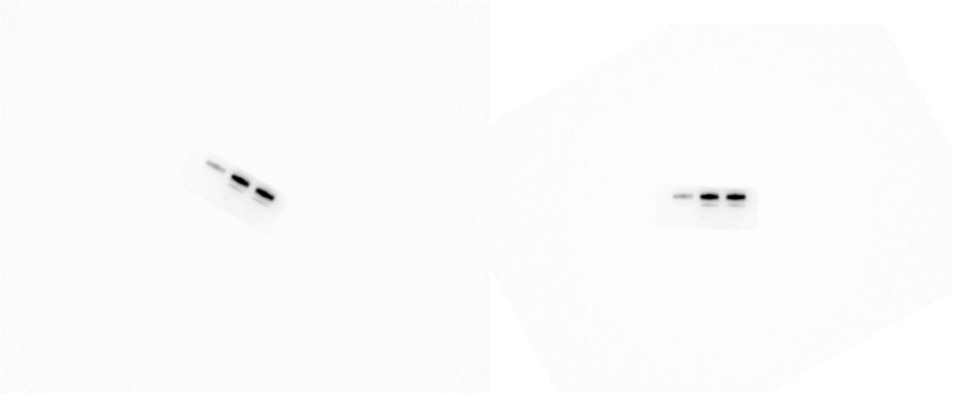

Figure4 B

GAPDH(1,2)

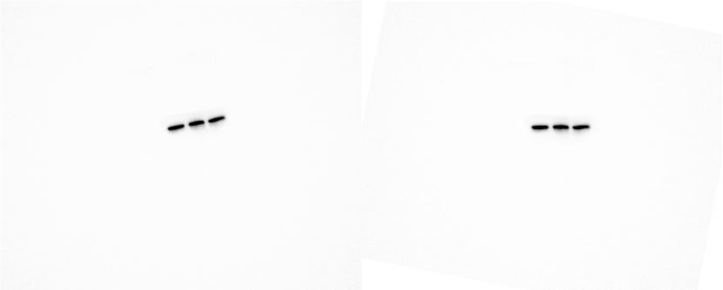

Flag

myc

Figure4 C

GAPDH(1,2)

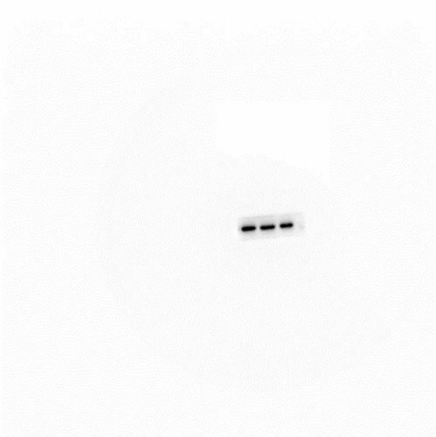

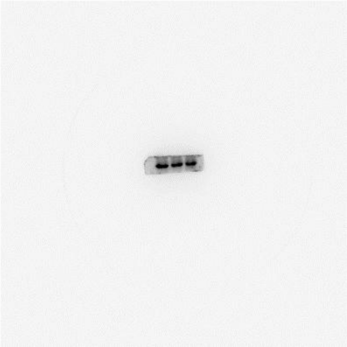

NRP1

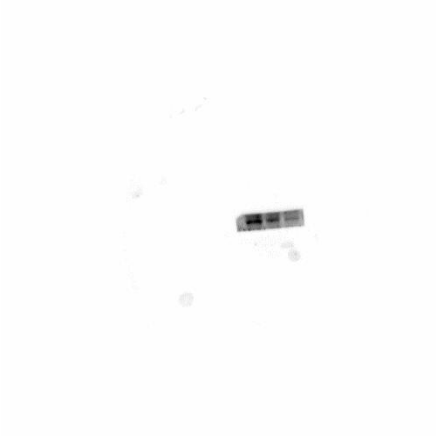

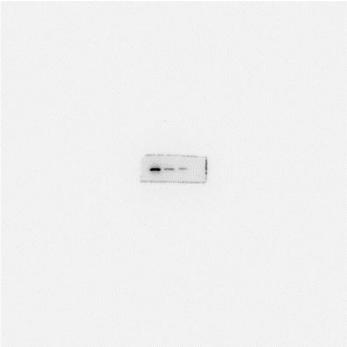

USP9X(1,2)

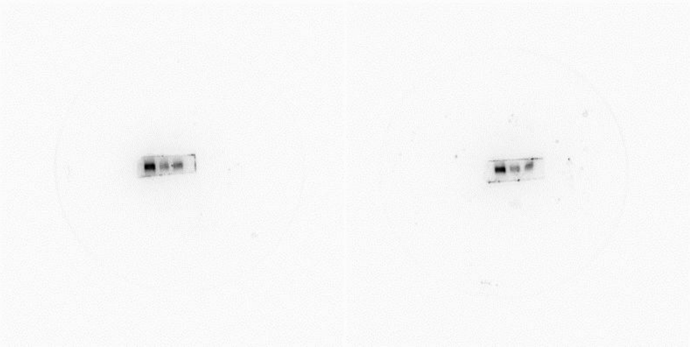

Figure4 E

GAPDH(1,2)

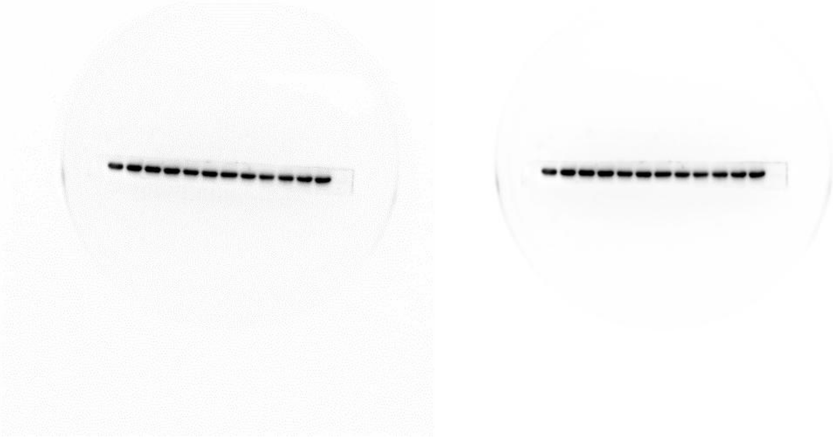

Nrp1(1,2)

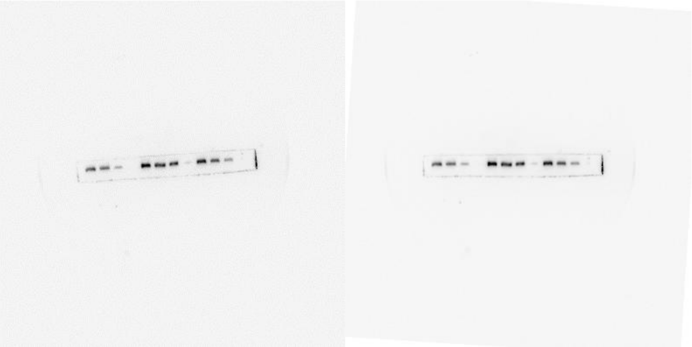

Usp9x(1,2)

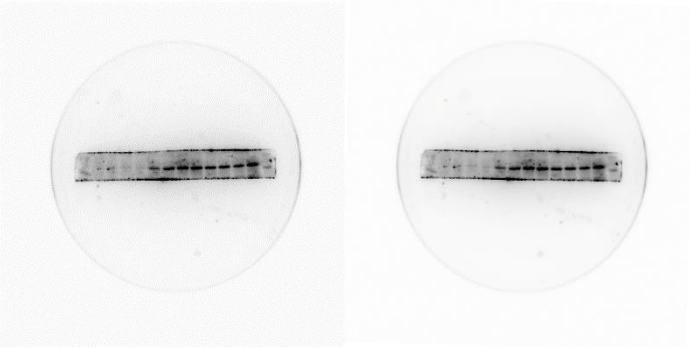

Figure4 F

GAPDH(1,2)

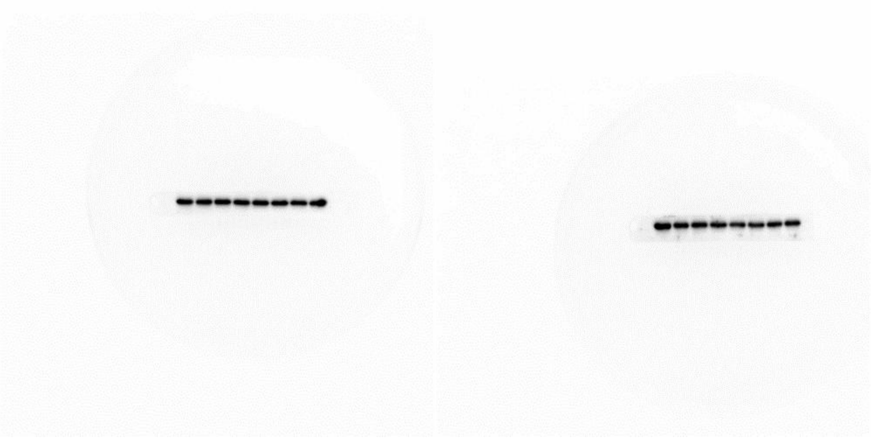

Nrp1(1,2)

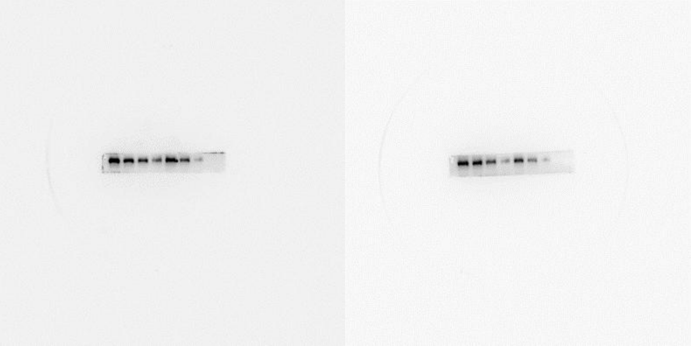

Nrp1(11,22)

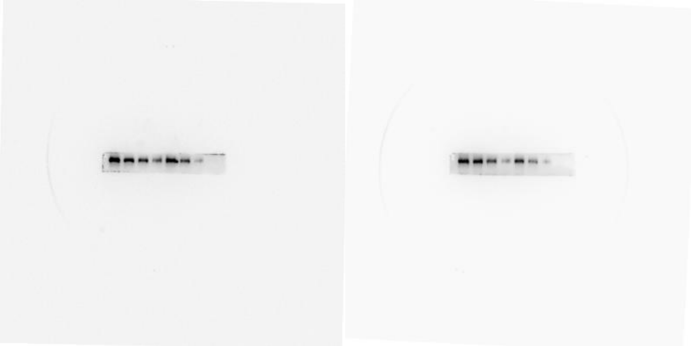

Usp9x(1,2,11)

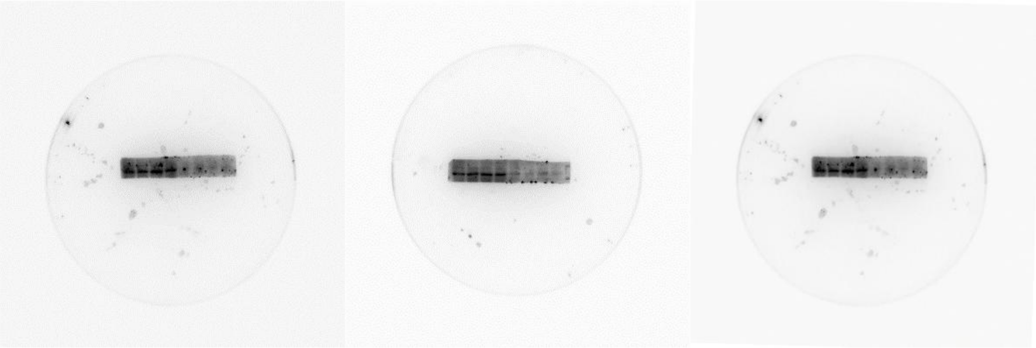

Supplement: Supplementary file 5 — Original Data File [file 41419_2022_5527_MOESM5_ESM.pdf]
