# Supplementary material for: USP9X-mediated NRP1 deubiquitination promotes liver fibrosis by activating hepatic stellate cells
Source: Cell Death Dis. 2023 Jan 19;14(1):40. doi: 10.1038/s41419-022-05527-9 (PMC9849111; doi:10.1038/s41419-022-05527-9)

Figure A1

GAPDH

antimyc

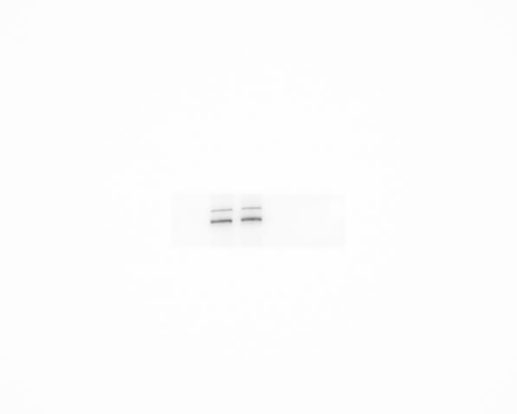

Ibflag(1,2)

IBHA(1,2)

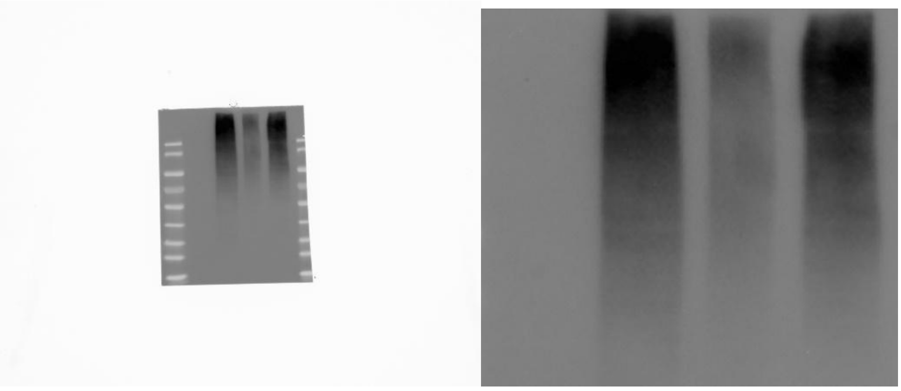

Figure A2

GAPDH(1,2)

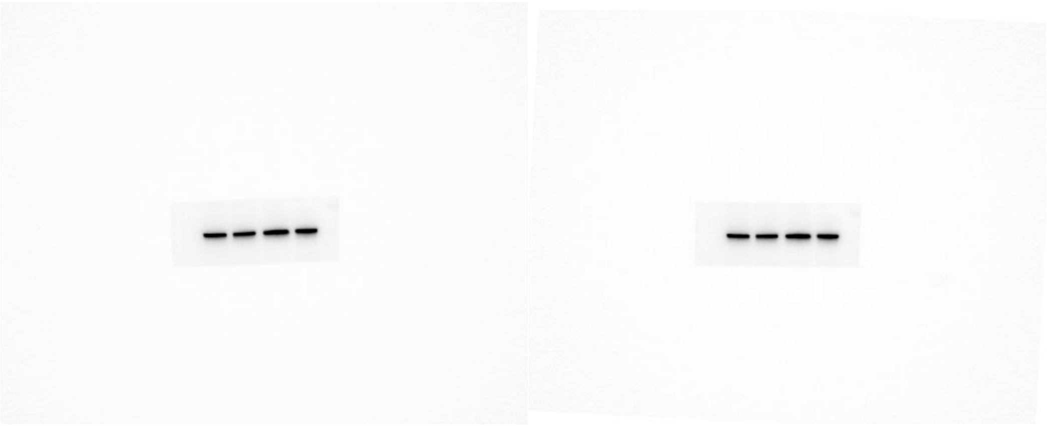

Antimyc(1,2)

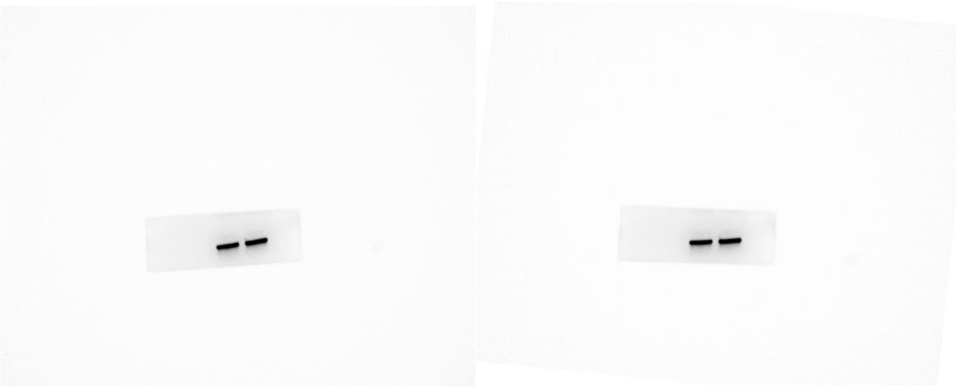

Ibflag(1,2)

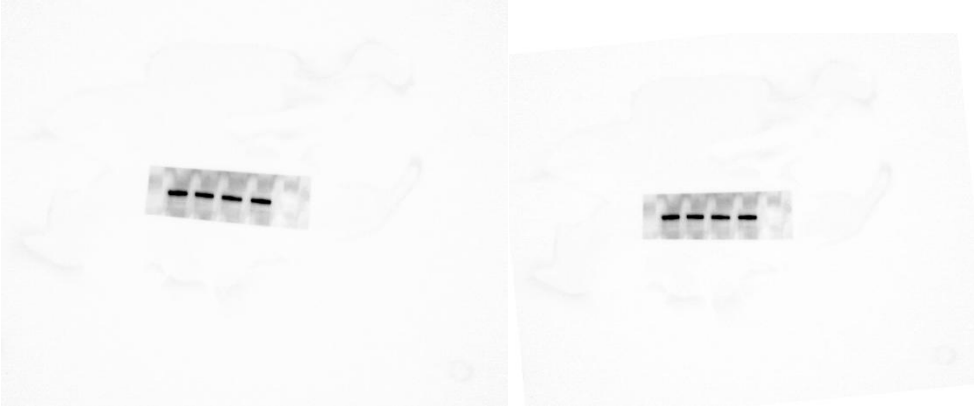

Ibha（1,2）

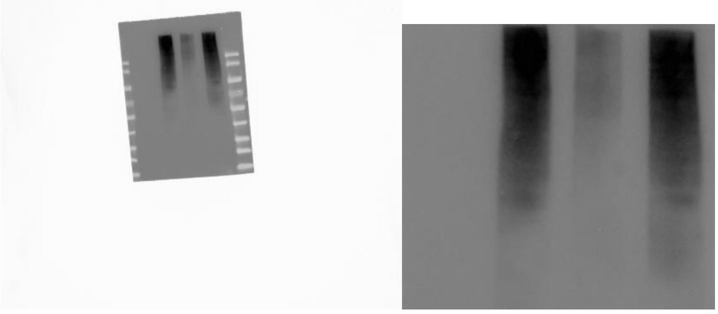

Figure B1

GAPDH

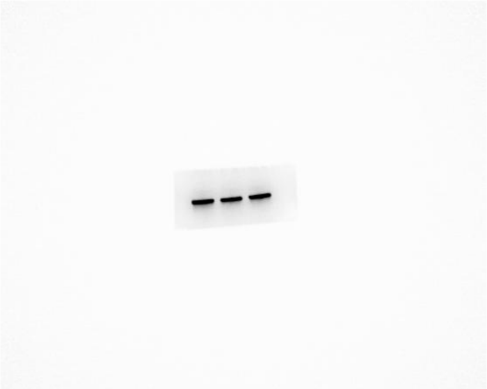

IBHA

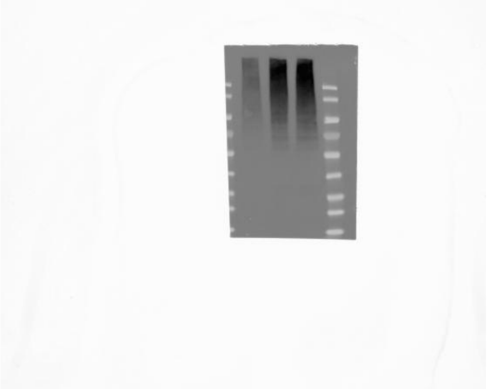

Ibnrp1

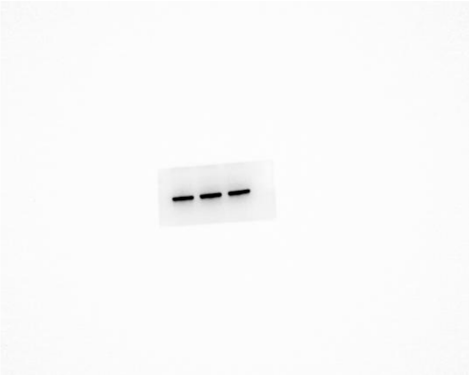

Usp9x

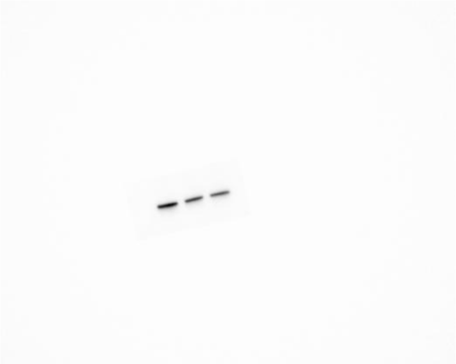

Figure B2

GAPDH

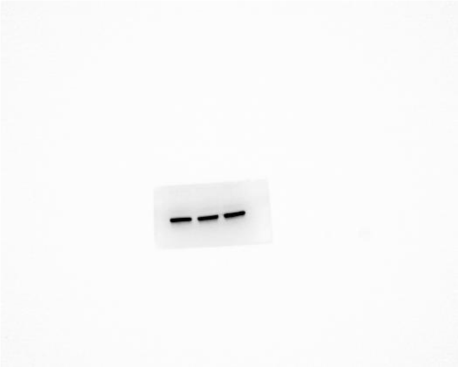

IBHA

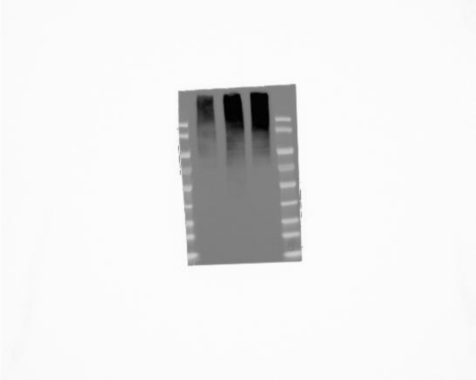

Ibnrp1

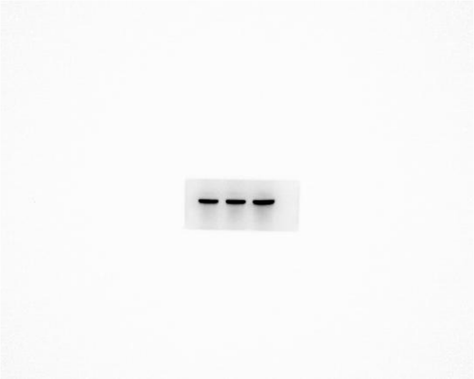

Usp9x

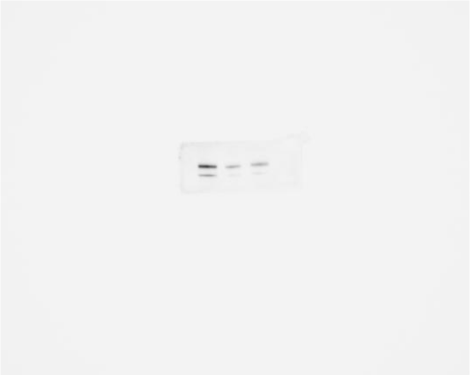

Figure C

Antiflag(1,2)

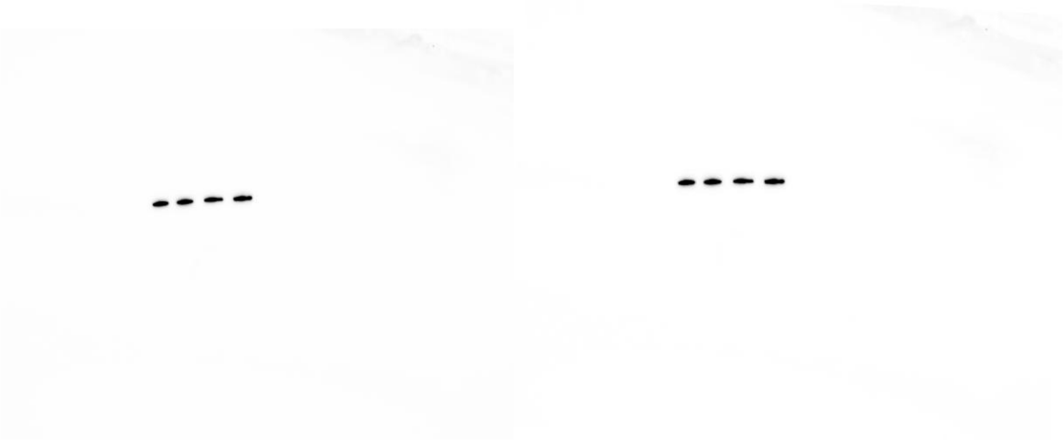

Ibflag(1,2)

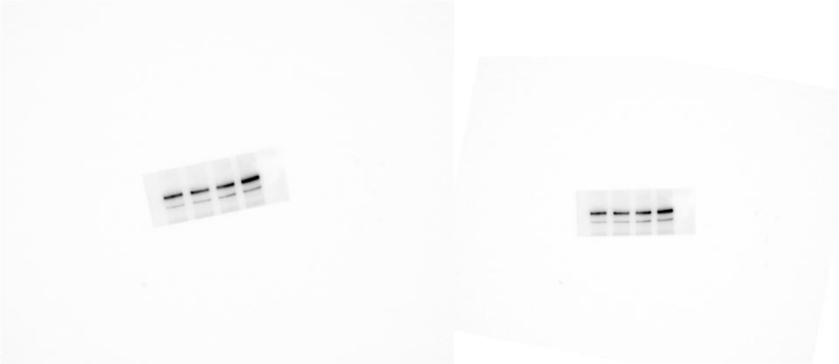

Ibha(1,2)

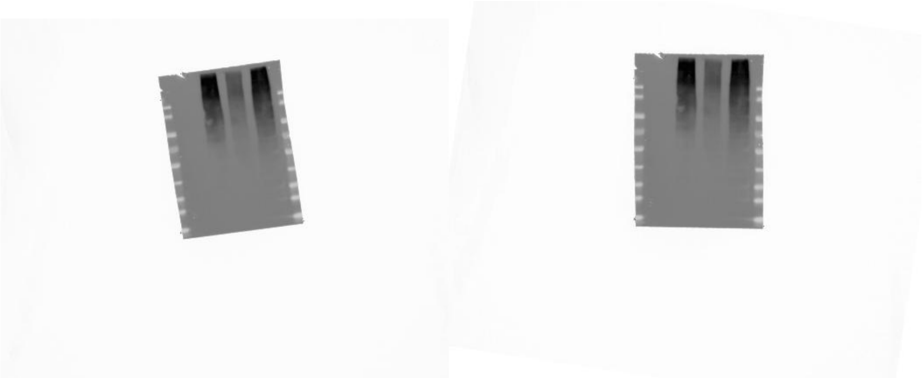

Usp9x(1,2)

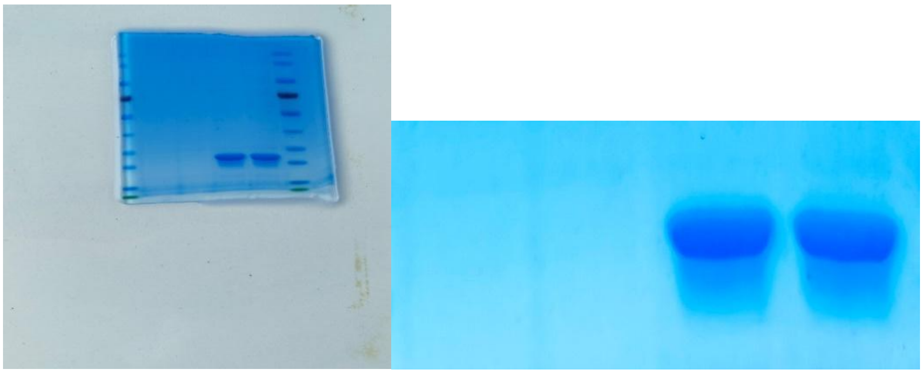

FigureD

GADPH(1,2)

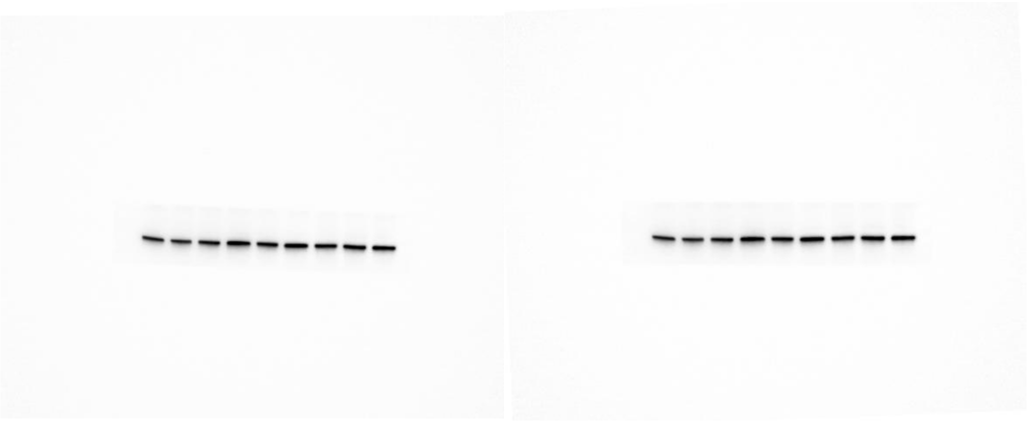

Antimyc(1,2)

Ibflag(1,2)

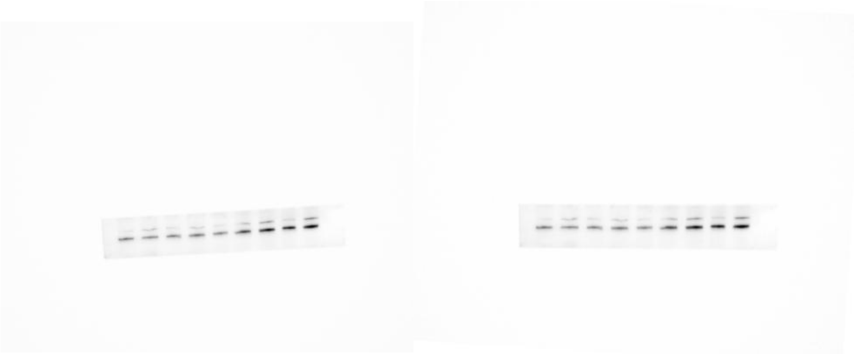

Ibha(1,2)

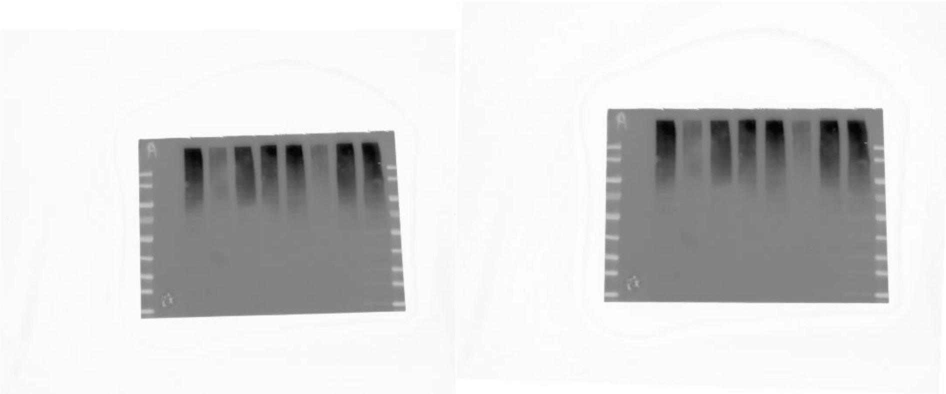

Figure E

GAPDH

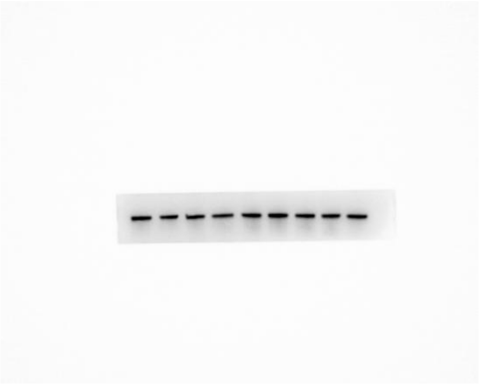

Antimyc(1,2)

ibflag

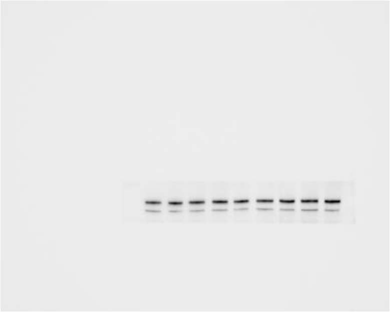

ibha

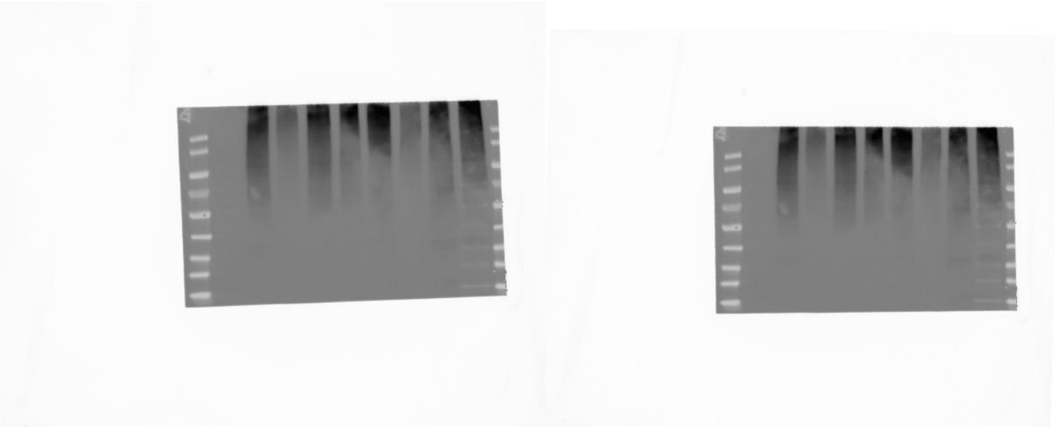

Supplement: Supplementary file 6 — Original Data File [file 41419_2022_5527_MOESM6_ESM.pdf]
