# Supplementary figures and images for: USP9X-mediated NRP1 deubiquitination promotes liver fibrosis by activating hepatic stellate cells
Source: Cell Death Dis. 2023 Jan 19;14(1):40. doi: 10.1038/s41419-022-05527-9 (PMC9849111; doi:10.1038/s41419-022-05527-9)

**B**

GAPDH

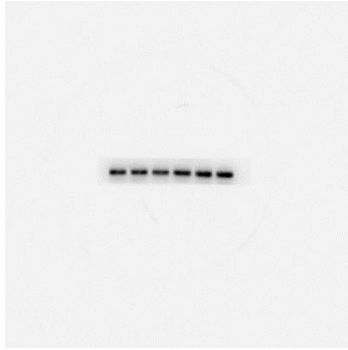

USP9X

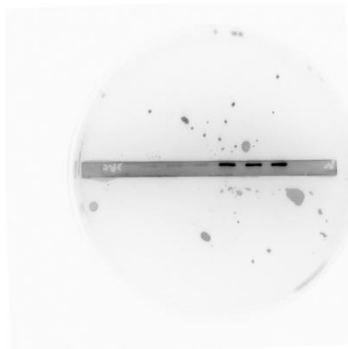

**Figure 6C**

GAPDH

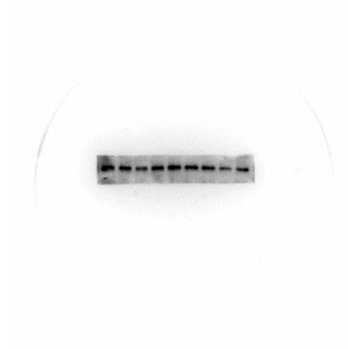

$\alpha$ -SMA

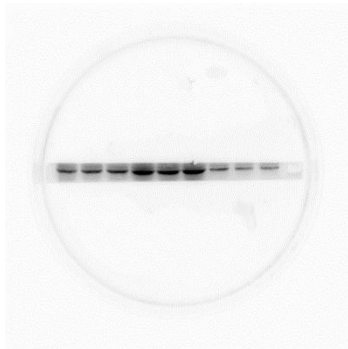

collagen I(1,2)

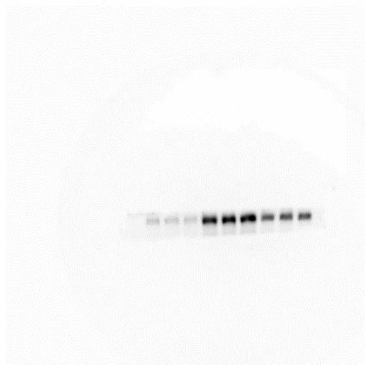

NRP1

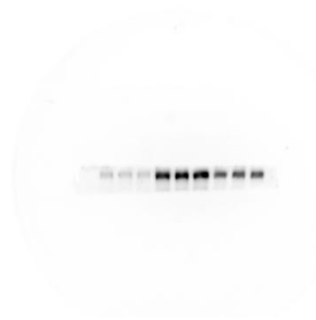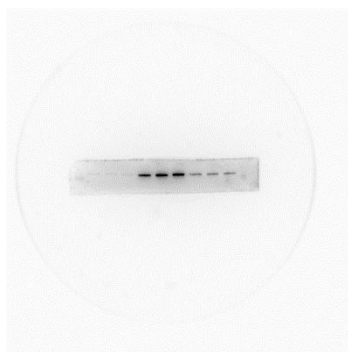

USP9X

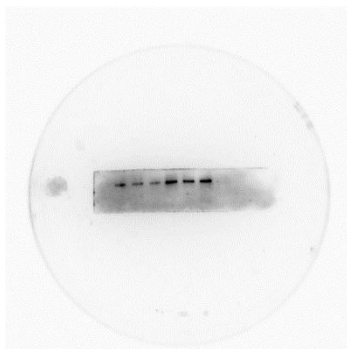

H

GAPDH

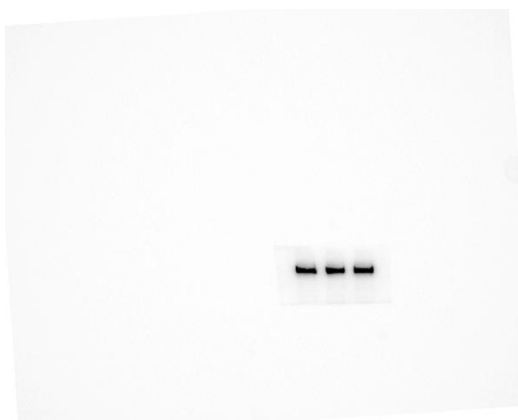

$\alpha$ -SMA

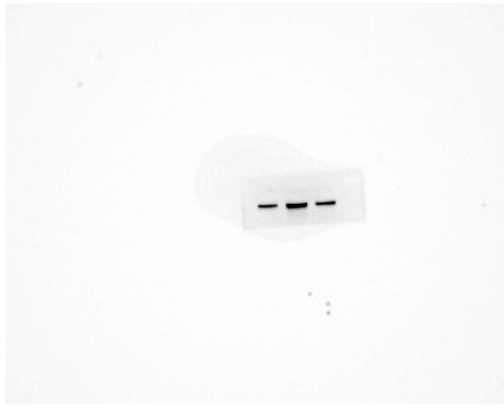

collagen I

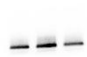

NRP1

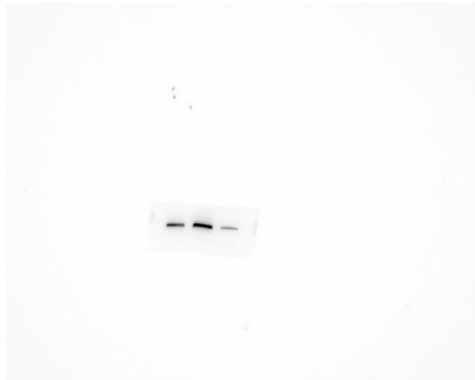

USP9X

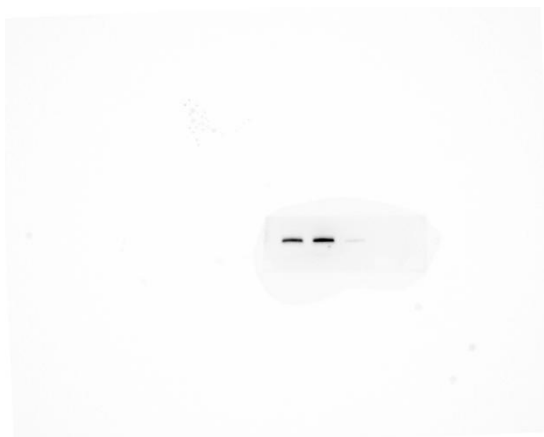

Supplement: Supplementary file 7 — Original Data File [file 41419_2022_5527_MOESM7_ESM.pdf]

GAPDH 1-2-1

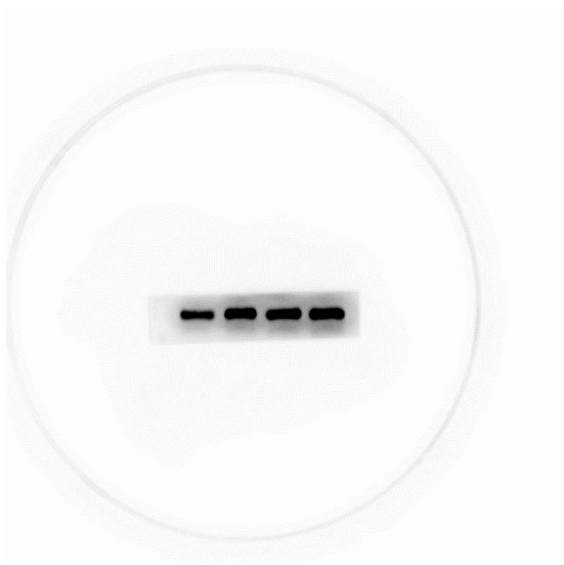

NRP1

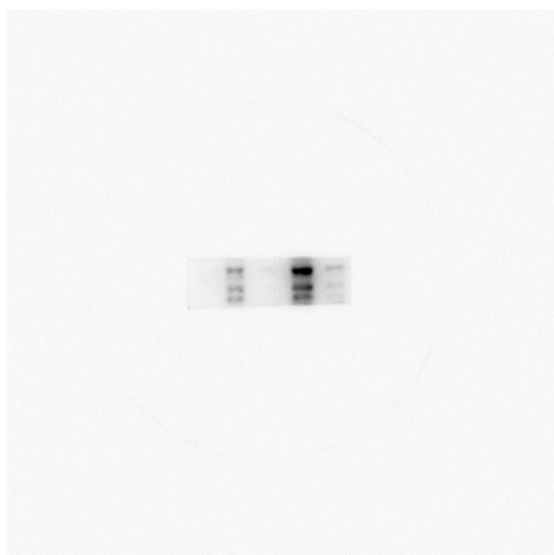

Supplement: Supplementary file 8 — Original Data File [file 41419_2022_5527_MOESM8_ESM.pdf]

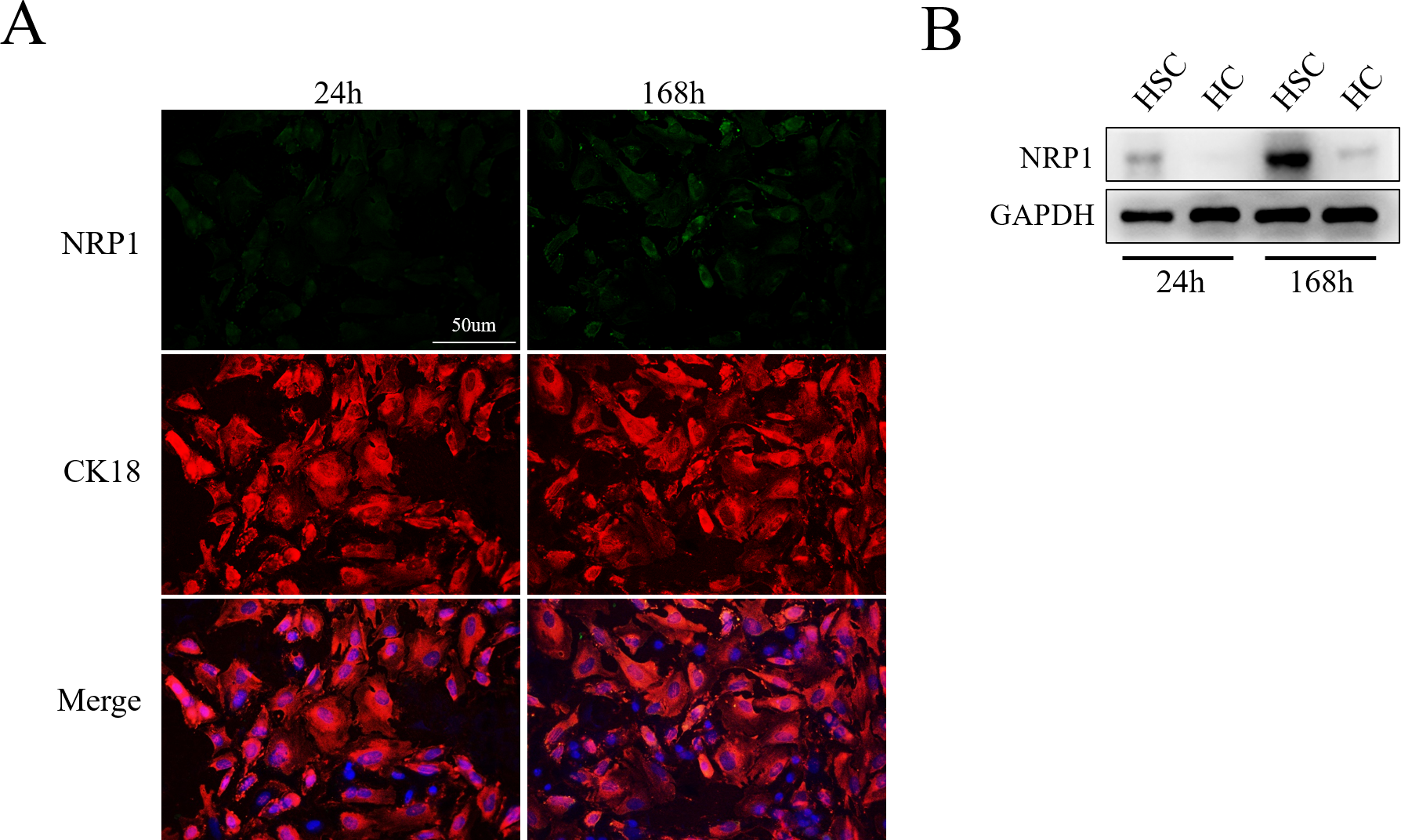

Supplement: Supplementary file 9 — SUPPLEMENTAL figure1 [file 41419_2022_5527_MOESM9_ESM.tif]
