# Supplementary material for: USP9X-mediated NRP1 deubiquitination promotes liver fibrosis by activating hepatic stellate cells
Source: Cell Death Dis. 2023 Jan 19;14(1):40. doi: 10.1038/s41419-022-05527-9 (PMC9849111; doi:10.1038/s41419-022-05527-9)
Supplement: Supplementary file 10 — EDITORIAL CERTIFICAT [file 41419_2022_5527_MOESM10_ESM.pdf]

https://www.mjeditor.com

EDITORIAL CERTIFICATE

This document certifies that the manuscript listed below was edited for grammar,punctuation,spelling,and overall style by one or more expert native English speaking editors with a PhD degree.

Manuscript information

ID：MJ24448

Editing date：2022.11.14

Title :USP9X-mediated NRP1 deubiquitination promotes liver fibrosis by activating hepatic stellate cells

Author(s): Jie Bai, Jinqiu Zhao, Fengling Peng, Chan Qiu, Yongguo Li, Li Zhong.

Language writing before editing: ☐Very poor ☐Poor ☑Fair ☐Good ☐Very good ☐Excellent

Recommendation after language editing:
☑Submitting to target journal directly
☐Submitting to target journal after minor revision
☐Re-editing required after major revision
☐Not suitable for publication

Certificate by

Sophiya.K

Editor in Chief
MJ Language Editing Services,Shenzhen,China

Disclaimer:Our service does not involve authenticity review or ethical review on the data(including images)presented in the manuscript.Neither the research content nor the author's intentions were altered in any way during the editing process.Documents receiving this certification should be English-ready for publication.The authors have the option to accept or reject our suggestions and changes in the edited document.However,we do not bear responsibility for revisions made to the document after our editing.If the manuscript is suspected of plagiarism,please contact the authors in time.

MJ Language Editing Services
Diwang Building, No. 5002 Shennan Road, Luohu District, Shenzhen, China
Tel:+086 0755 25100506
